# Clinical evidence for overcoming capecitabine resistance in a woman with breast cancer terminating in radiologically occult micronodular pseudo-cirrhosis with portal hypertension: a case report

**DOI:** 10.1186/1752-1947-4-112

**Published:** 2010-04-21

**Authors:** Christa Fournier, Glenn Tisman, Robert Kleinman, Yong Park, William D MacDonald

**Affiliations:** 1 Glenn Tisman, M.D. Medical Corporation, Bailey Street, Whittier, California, 90601, USA; 2 Radiology Department, Presbyterian Intercommunity Hospital, Washington Boulevard, Whittier, California, 90602, USA; 3 Pathology Department, Presbyterian Intercommunity Hospital, Washington Boulevard, Whittier, California, 90602 USA

## Abstract

**Introduction:**

We report a case of stage IV breast cancer terminating in an unusual picture of radiologically occult micronodular pseudo-cirrhosis. Contrast-enhanced computed tomography showed no evidence of metastatic breast cancer within the liver. Unlike the few previously reported cases of intrasinusoidal spread of breast cancer, our patient was palliated with a transjugular intrahepatic portosystemic shunt along with salvage chemohormonal therapy. In addition, our patient demonstrated proof of the principle of the dependence of capecitabine (Xeloda) efficacy on dose scheduling as predicted by laboratory studies based on Gompertzian tumor growth and the Norton-Simon hypothesis.

**Case presentation:**

We report the case of a 52-year-old Caucasian woman who developed radiological signs of portal hypertension without radiological evidence of hepatic metastasis five years after being diagnosed with metastatic breast cancer. She was receiving chemotherapy for stage IV breast cancer initially thought to be metastatic only to the bones. During salvage therapy with high-dose estradiol (Estradiol valerate), vinorelbine (Navelbine) and bevacizumab (Avastin), she suddenly developed signs of portal hypertension confirmed on computed tomography and by portal and systemic venous pressure measurements. Drug toxicity due to bevacizumab (Avastin) was initially and incorrectly entertained as a cause. The patient underwent palliative transjugular intrahepatic portosystemic shunt and transhepatic venous liver biopsy, which revealed the presence of rapidly progressive and uncontrolled metastatic breast cancer. The new discovery of radiologically occult intrasinusodal hepatic metastases with secondary micronodular cirrhosis was found to be the cause of her sudden onset portal hypertension. The patient's resistance to capecitabine (Xeloda) was reversed by changing the schedule of medication to biweekly 7/7 (7 days ingesting drug alternating with 7 days off drug) from the 14/7 (14 days ingesting drug alternating with a 7 day rest period) day schedule approved by the US Food and Drug Administration.

**Conclusion:**

This case report demonstrates an unusual presentation of radiographically occult hepatic metastasis from breast cancer palliated with transjugular intrahepatic portosystemic shunt. All patients with advanced breast cancer developing unexpected portal hypertension should be considered candidates for liver biopsy despite normal computed tomography of the liver imaging results. This is the first report of a reversal of clinical resistance to capecitabine (Xeloda) by changing from the schedule of 14/7 day to a biweekly 7/7 day schedule. This suggests that a biweekly schedule may be best for some patients.

## Introduction

We describe a patient who underwent extensive treatment for stage IV breast cancer that had metastasized to her bones and liver. She had unique and medically important clinical features of radiologically occult liver metastases and drug resistance to schedule-dependent chemotherapy. Contrast-enhanced computed tomography (CT) did not demonstrate metastatic liver disease, although it did show advanced portal hypertension.

Three similar cases and a review of English literature by Allison *et al*. confirmed the rarity of such a presentation (21 reported cases) [1]. Most of the patients previously reported were not treated with transjugular intrahepatic portosystemic shunt (TIPS). Our patient underwent a TIPS procedure, which rendered a modest relief of her symptoms. Meanwhile, secondary hepatic encephalopathy did not occur. It was apparent that our patient's drug resistance to capecitabine (Xeloda) was reversed by changing drug scheduling. Such had been previously predicted by laboratory experiments, thus lending support to the validity of chemotherapy schedule modelling based on Gompertzian tumor cell kinetics and the Norton-Simon hypothesis [2-5].

## Case presentation

A 52-year-old Caucasian woman was diagnosed at age 47 with Stage IIB, Grade II T2N3 Mx invasive ductal adenocarcinoma of the breast. She had 14 of 18 tumor positive nodes, wherein one had capsular penetration. Examination showed the following values for our patient: estrogen receptor = 69%, progesterone receptor = 27%, Ki67 = 10% and HER2/neu was not over expressed. She then underwent mastectomy. She was treated within one month with adjuvant chemotherapy. For five months our patient received dose-dense sequential hydroxydaunomycin (Doxorubicin), paclitaxel (Taxol) and cyclophosphamide (Cytoxan). She received four applications of each drug once every 14 days.

After completing radiation therapy, she was started on exemestane (Aromasin) and leuprolide (Lupron) for the next nine months. On the eighth month, her CA 27 to 29 increased to 47 U/ml. Bone imaging confirmed osseous metastases. At that time she elected not to receive intravenous chemotherapy. Her relapse after two years was treated with metronomic low-dose oral cyclophosphamide (Cytoxan) and methotrexate (Trexall) plus tamoxifen (Soltamox). She showed no response to this medication. She subsequently began monthly fulvestrant (Faslodex) and weekly albumin-bound paclitaxel nanoparticles (Abraxane).

**Figure 1:**
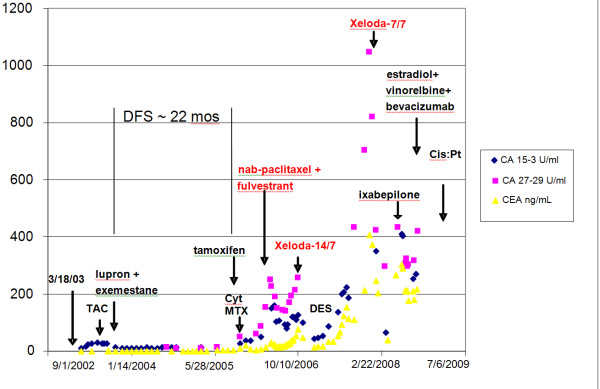
**The patient's tumor markers over time since her breast cancer was diagnosed**.

After a 5-month response, which was then followed by resistance, these drugs were discontinued following a total of 17 albumin-bound paclitaxel nanoparticles infusions (Abraxane). She then complained of a new, diffuse and severe bone pain. A bone scan confirmed that diffuse bony metastases had progressed. This was associated with a rise in her CEA from 37.2 to 75.1 ng/ml, CA 15-3 from 88 to 128 U/ml and CA 27-29 from140 U/ml to 285 U/ml. From 16 October 2006 to 15 April 2007, she was treated with daily capecitabine (Xeloda) 1500 mg in the morning and 1000 mg before bedtime two weeks on and one week off as per the US Food and Drug Administration (FDA) approved 14/7 21-day cycle. This was her maximally tolerated dose. She had severe diarrhea limited by gastrointestinal toxicity when trying to increase the dose of her medicaiton by an additional 500 mg/day. She developed a 6-month tumor marker (CEA and CA 15-3) and clinical response with complete relief of disabling diffuse bone pain and tumor marker analysis as outlined in Figure 1.

Subsequently, her tumor pain and markers increased and 2 mg diethylstilbestrol compounded (DES) was administered twice daily from 18 April 2007 to 17 June 2007. This treatment was judged to be ineffective.

She was rechallenged with capecitabine (Xeloda) and the schedule was changed to the biweekly 7 days on, 7 days off regimen based on experiments by Dr. Larry Norton on the benefits of biweekly capecitabine (Xeloda) [5]. From 26 November 2007 to 03 June 2008, she took capecitabine (Xeloda) 2000 mg orally twice daily one week on and then followed by one week off. This dosing schedule change was associated with a new durable clinical and laboratory remission that lasted approximately six months. Her CA 27-29 decreased from 1025 U/ml to 325 U/ml, while her CA 15-3 decreased from 325 U/ml (<35 U/ml) to 42 U/ml and her CEA from and 410 ng/ml (<5 ng/ml)) to 13.8 ng/ml. A summary of her tumor marker response is presented in Figure 1.

Our patient's clinical response was characterized by complete relief from diffuse bone pain which was similar to her first capecitabine (Xeloda) response. However, on 18 June 2008, due to the recurrence of her intense bone pain and increasing tumor markers (CEA from 13.8 to 220 ng/ml) she patient was withdrawn from capecitabine (Xeloda) and given a single dose of ixabepilone (Ixempra). She showed no response to this medicaiton. As a result, bevacizumab (Avastin) was added every 14 days from 19 June 2008 to 10 September 2008 with a total of five doses. On 14 August 2008, she began weekly vinorelbine (Navelbine). She was also started on weekly injections of 60 mg estradiol velestrate on 21 August 2008.

**Figure 2:**
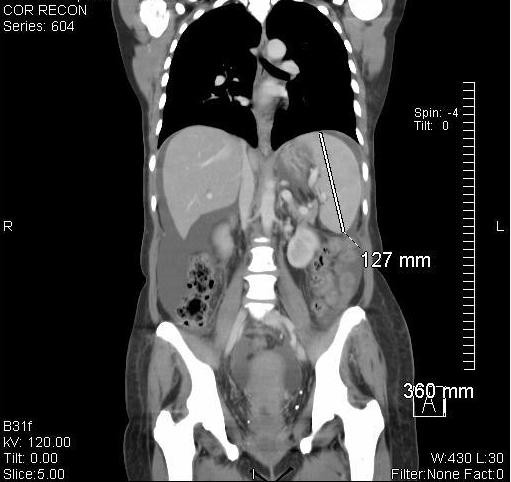
**Splenomegly, ascites and normal appearing hepatic parenchyma**.

**Figure 3:**
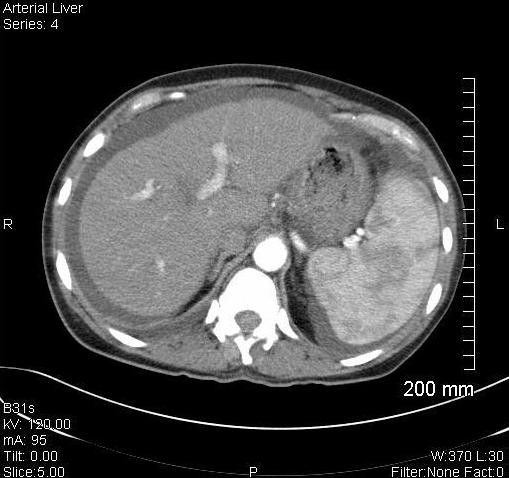
**Splenomegly, ascites, esophageal varacies and normal appearing hepatic parenchyma**.

During the month of October 2008, our patient complained of abdominal fullness and pressure discomfort of her upper abdomen. She had no history of hepatitis, cirrhosis, illicit drug or excessive alcohol ingestion, although she had nausea and loss of appetite. Blood studies revealed the following values: SGOT = 166 (<42), SGPT = 232 (<40), GGT = 161(<24), total bilirubin = 0.8 (<1.2), white blood cell = 8320, hematocrit = 24.7%, mean corpuscular volume = 86.6 and platelets = 50,000/mm^3^. A metastatic evaluation including bone X-rays, contrast enhanced CT of the chest, abdomen and pelvis, as well as CT angiogram of her lungs was completed. The studies revealed no evidence for hepatic metastases, an absence of pulmonary emboli, and the presence of diffuse osteoblastic bone metastases. There was unexpected ascites, splenomegaly and esophageal varacies suggestive of portal hypertension (Figures 2 and 3). Because of the progressive ascites, splenomegaly, abdominal discomfort and nausea, along with presumed hypersplenic sequestration of platelets, which limited chemotherapy options, our patient underwent a TIPS procedure with concomitant diagnostic transhepatic venous liver biopsy.

A portal venogram confirmed the presence of esophageal varacies in our patient. Pre-TIPS portal venous pressure measured 31 mmHg, while her central venous pressure was at 8 mmHg. Her portal systemic gradient was 23 mmHg (normal<10). Post-TIPS the portal-systemic gradient decreased to normal = 3 mmHg. This was associated with a rapid shift of ascitic fluid into the systemic circulation with transient pulmonary edema and dyspnea on the day of the procedure. Encephalopathy did not occur. After a rapid and almost complete resolution of her ascitic accumulation our patient was significantly relieved of her abdominal discomfort. Her platelet count only slightly increased from 50 to 70,000 a week after the TIPS procedure.

**Figure 4:**
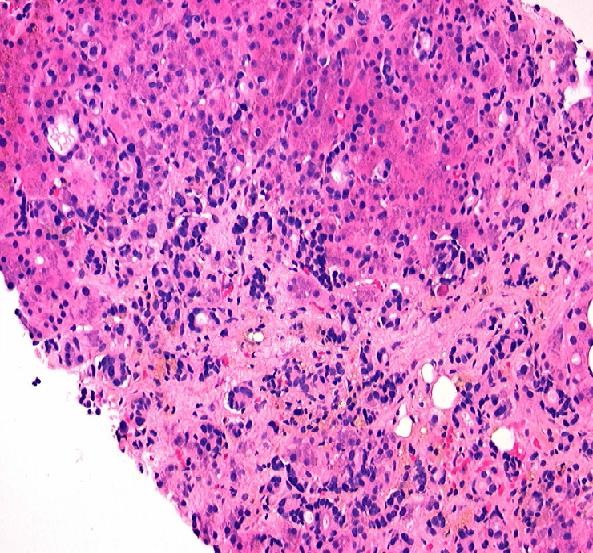
**200× magnification of photomicrograph of liver parenchyma showing micronodular cirrhotic changes and extensive infiltration by metastatic breast cancer cells**.

**Figure 5:**
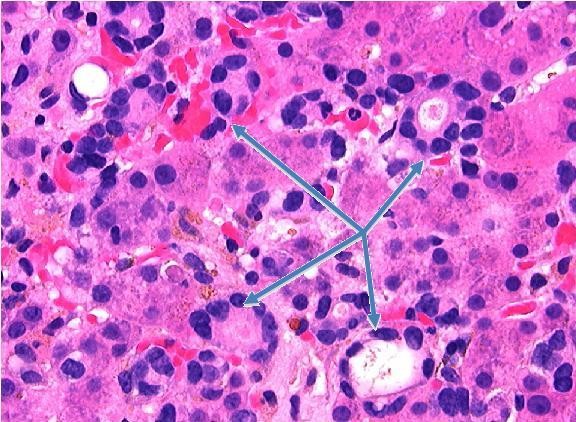
**630× magnification of photomicrograph showing metastatic breast carcinoma cells with distinct ductal histoarchitecture infiltrating liver sinusoids (arrows)**.

**Figure 6:**
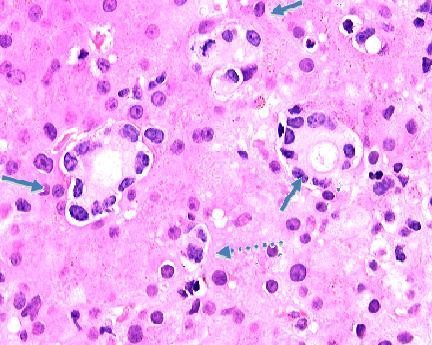
**630× magnification of photomicrograph showing metastatic breast carcinoma cells infiltrating the liver sinusoids**. Note the duct-like structures (arrows) with lining cells that have lost nuclear polarity and the mitotic form (dashed arrow).

**Figure 7:**
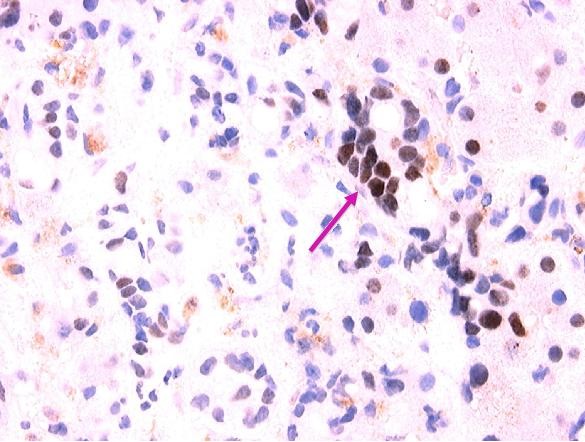
**630× magnification of photomicrograph showing the results of immunohistochemical staining for the estrogen receptor**. Note the metastatic, estrogen receptor positive breast carcinoma cells infiltrating the liver sinusoids (arrow).

Meanwhile, histopathology revealed the true nature and cause for our patient's symptoms and portal hypertension. Biopsy revealed the unusual pattern of micronodular cirrhosis induced by a diffuse intrasinusoidal infiltration of moderately-differentiated breast cancer cells. The cells biopsied were HER-2/neu negative, with estrogen receptor (ER) at 7% and progesterone receptor (PR) at 3%. The original tumor prior to any therapy was marked with an ER of 69%, PR of 27%, and negative HER-2/neu negative. Metastatic breast carcinoma cells with distinct ductal histoarchitecture were diffusely infiltrating our patient's liver sinusoids (Figures 4, 5, 6, 7), which was similar to the cases presented by Allison *et al*. [1]. Our patient's ascitic fluid contained morphologically similar tumor cells. She continued a rapid downhill course with her total bilirubin increasing to 17 mg/dl. A last effort using the administration of infusional cisplatinum (Cisplatin) 25 mg/m2/d for 5 days was associated with a slight decrease in all her tumor markers, although this lasted only for days. The patient and her family subsequently requested hospice care. A graph showing our patient's tumor markers since she was diagnosed with breast cancer, along with the dates she began different treatments, is shown in Figure 1.

## Discussion

Our patient demonstrates two important and unique features involving the treatment and histopathology of breast cancer metastases. The fact that she was able to enjoy an additional six months of capecitabine (Xeloda) control of painful bone metastases was due to a change in dosing schedule, the biological basis of which was provided by the Norton-Simon model. This model, which was based on Gompertzian tumor growth, suggests that by increasing the dose density of chemotherapy during the rapid tumor growth phase, greater cell kill can be induced. Enhanced cell kill is thus obtained through a greater chemotherapy dose rate. Such a strategy ideally limits the regrowth of tumor cells between cycles of chemotherapy [2-5].

The pioneering work by Norton *et al*. on capecitabine (Xeloda) scheduling motivated the schedule change for our patient [2-6]. The biweekly 7/7 capecitabine schedule delivered 28,000 mg of capecitabine over 14 days compared to the FDA-approved 14/7 protocol that delivered 35000 mg over 14 days every 21 days. One difference is that 28000 mg of capecitabine delivered in the 7/7 schedule was ingested over 7 days (dose density = 28000 mg/7d) while the 35000 mg dose of the 14/7 schedule was delivered over 14 days (dose density = 17500 mg/7d). Compared to the 14/7 schedule the 7/7 biweekly schedule delivered 1.6 times the dose of capecitabine over the 7-day period of drug administration. The 14/7 schedule was the maximum tolerable dose for our patient because of her gastrointestinal toxicity, while the 7/7 schedule had no such toxicity. This benefit was as predicted by the experiments outlined in Norton's work [6].

The second feature is of occult liver metastases due to breast cancer. Most liver metastases from breast cancer are apparent on contrast-enhanced CT imaging and rarely cause portal hypertension and progressive liver failure. Allison *et al*. described three cases of breast cancer metastatic to the liver in the absence of CT detection [1]. The livers in their patients were homogeneous and contained no discrete metastatic lesions. Only one of their patients had portal hypertension by hepatic ultrasound. Microscopically, as noted in our patient, there were moderately-differentiated tumor cells diffusely infiltrating throughout the hepatic sinusoids [1]. Allison *et al*. concluded that intrasinusoidal hepatic metastases of breast carcinoma can occupy a large percentage of the hepatic volume, yet remain occult both radiographically and macroscopically. Their patients presented with advanced liver failure. They mentioned additional breast cancer patients presenting with diffuse intrasinusoidal hepatic metastases (all invasive ductal subtypes) as a result of their review of the English literature where 9 of 18 had no radiological evidence of metastatic disease [1].

There is a previously described radiological entity termed "pseudo-cirrhosis" due to breast metastases treated with chemotherapy [7,8]. This entity is the result of a reaction to chemotherapy of visible hepatic metastases which results in the radiological appearance of macronodular cirrhosis. Our case is unique because of the unusual pattern of tumor cell infiltration and the peculiar micronodular cirrhotic response that remained radiographically occult within our patient's liver parenchyma while causing radiologically advanced changes of portal hypertension including splenomegaly, esophageal varacies, ascites and thrombocytopenia (the etiology of which was initially confused with bone marrow infiltration by tumor and or chemotherapy effect).

## Conclusion

This case report presents evidence for the superiority of a 7/7 day capecitabine [Xeloda] dosing schedule. This is the first clinical demonstration of the 7/7 regimen's superiority where the patient acted as her own control. The superiority of the 7/7 regimen was predicted in the work of Norton *et al*. based on the Norton-Simon hypothesis and on animal studies where the maximal affect of capecitabine occurs about 8 days after the initiation of therapy. Drug delivered after that period (days 8 to 14) had a higher toxicity-to-benefit ratio [6].

Medical oncologists depend extensively on the use of contrast-enhanced CT imaging to rule out hepatic metastases. Although isodense tumor metastases are well described, their presentation as portal hypertension with rapid progressive liver failure is unusual. Patients with breast cancer who suddenly develop signs and symptoms of portal hypertension in the absence of contrast-enhanced CT imaging, hepatic abnormalities will require biopsy to rule out liver metastases.

## Consent

Written informed consent was obtained from the patient for publication of this case report and any accompanying images. A copy of the written consent is available for review by the Editor-in-Chief of this journal.

## Competing interests

The authors declare that they have no competing interests.

## Authors' contributions

CF performed the basic research for the article and wrote portions of the manuscript. GT was the patient's medical oncologist, provided the conceptual design of the case report, and wrote the majority of the manuscript. RK provided diagnostic radiology and interpretation. YP performed and interpreted the results of the transjugular intrahepatic portosystemic shunt procedure. WM was responsible for pathological diagnostics and provided the photomicrographs of the liver pathology. All authors read and approved the final manuscript.
